# *Lindera pulcherrima* var. *attenuata* leaves: a nutritious and economically promising staple food in the Baiku Yao community in China

**DOI:** 10.3389/fnut.2023.1193328

**Published:** 2023-05-18

**Authors:** Binsheng Luo, You Nong, Renchuan Hu

**Affiliations:** ^1^Lushan Botanical Garden, Jiangxi Province and Chinese Academy of Sciences, Lushan, China; ^2^Guangxi Key Laboratory of Traditional Chinese Medicine Quality Standards, Guangxi Institute of Traditional Medical and Pharmaceutical Sciences, Nanning, China

**Keywords:** *Lindera pulcherrima* var. *attenuata*, nutrition, ethnobotany, food plant, Baiku Yao

## Abstract

The Baiku Yao community in China has traditionally used the *Lindera pulcherrima* var. *attenuate* (LPA) leaves as a staple food source, diverging from the common reliance on grains and plant roots worldwide. This study comprehensively analyzes LPA’s nutritional composition and local usage practices through field observations and laboratory testing. Our results show that LPA contains significant levels of starch, protein, and dietary fiber and is rich in trace elements, vitamin E, and flavonoids. These findings not only support the community’s traditional knowledge of LPA as a valuable food and feed source but also highlight its potential as a sustainable and innovative ingredient for new food and feed products. By filling a gap in our scientific understanding of LPA, this study may contribute to developing novel and eco-friendly agricultural practices and promote regional economic growth.

## 1. Introduction

Starch-based plant foods, such as cassava, yam, rice, and wheat, have long been mostly relied upon by human beings as a primary source of energy, derived from plant roots and seeds. As a result, food crops have been deemed strategic resources by different countries (1). Recently, a confluence of factors, including the COVID-19 pandemic, and extreme weather events, has led to decreased food production and disrupted food trade. Consequently, the issue of “food security” has gained increasing importance (2, 3). To address this challenge, researchers in the field of food safety are increasingly focused on exploring agricultural biodiversity and developing new plant-based foods (4).

Compared with urban communities that rely heavily on food trade, local communities tend to have more choices of energy and nutrition sources in the face of sudden disasters because of their reliance on agricultural production and high biodiversity (5). Collecting wild food plants can provide sufficient energy and nutrients for local community residents to make ends meet (6). The investigation of folk wild edible plants in different areas also provides many clues for new food crops (7). For example, in our investigation of the Hani rice terraces, we found that *Dioscorea subcalva* is one of the popular local food plants (8); due to its origin in the genus *Dioscorea*, it has a very high starch content and is often eaten as a staple food, and has been mentioned in ethnobotanical cases in some other regions (9, 10).

Generally, staple food plants worldwide predominantly rely on the underground components or seeds of plants, with limited documentation on the utilization of plant leaves as a staple food source. During a sequence of ethnobotanical studies conducted in the Baiku Yao region of China, certain informants in Baxu Township, Nandan County, revealed that the leaves of a green plant were consumed as a staple food during times of famine, which piqued our research interest. After sampling and identification, the plant is *Lindera pulcherrima* var. *attenuata* (LPA), an evergreen tree of the genus *Lindera* in Lauraceae, which are mainly produced in Guangdong, Guangxi, Hunan, Hubei, Yunnan, Guizhou, Sichuan, and other provinces and regions in China, often on hillsides and streams at an altitude of 65–1,590 m (11). At present, there is no research report on the nutrients or non-volatile active compounds contained in the LPA leaves, and there are few reports on the behavior of using the leaves of Lauraceae plants as a staple food. Therefore, this study intends to study the traditional folk use of LPA leaves and its nutritional components and total flavonoids, aiming to (1) discover, record, and protect its traditional knowledge and (2) evaluate the scientificity and development potential of related traditional knowledge.

## 2. Study method

### 2.1. Ethnobotanical research

In this study, by searching and reading the literature, we learned about the research status of LPA and collected its related traditional uses. We conducted an ethnobotanical interview on LPA in the Baiku Yao area to investigate the local use of LPA (12). The research site is Yaozhai Village, Baxu Township, Nandan County, Guangxi Zhuang Autonomous Region. The subjects of the interview are the elderly who are familiar with the folk use of LPA. The interview mainly uses the semi-structured interview method, and the content of the interview is to ask about the local distribution, collection, processing, and utilization of LPA. In addition, we also recorded the whole process of LPA processing through the method of participatory observation.

### 2.2. Nutrients and total flavonoids tests

The experimental samples were leaves of LPA collected from Yaozhai Village, Baxu Township, Nandan County, Guangxi Province, China, on December 20, 2021. We collected mature leaves from six populations of LPA, totaling approximately 1 kg, and mixed them together. Then, LPA was sun-dried after collection. The leaf nutrients of LPA were determined by different methods, including macroscopic nutrients, amino acids, vitamins, and some trace elements (Cu, Fe, Zn, Ca, Mg, K, Na, and P). The secondary metabolites, total flavonoid content, was also determined. The specific testing items and methods are shown in Table 1.

**Table 1:** Determination of nutritional components and total flavonoids in LPA.

Test items		Determination methods
Macronutrient	Energy	GB/Z 21922-2008
	Protein	GB 5009.5-2016 method 1
	Fat	GB 5009.6-2016 method 2
	Dietary fiber	GB 5009.88-2014
	Starch	GB 5009.6-2016 method 2
	Trace elements	GB 5009.268-2016 method 2
Vitamins	Vitamin A	GB 5009.82-2016 method 1
	Vitamin B1	GB 5009.84-2016 method 2
	Vitamin B2	GB 5009.85-2016 method 1
	Vitamin C	GB 5009.86-2016 method 2
	Vitamin E	GB 5009.82-2016 method 1
Total flavonoids		ISO 20759:2017(Annex C)

The determined methods mostly followed the *Standardization Technical Guidance Document of the People’s Republic of China*.

## 3. Results

### 3.1. The traditional use of LPA

Literature research shows that LPA’s branches, leaves, and bark contain aromatic oil and gum (11). In Guangxi, some people add leaf powder to pig feed to make it thicker and more fragrant; pigs like to eat it and can gain fat and the bark can be used as a medicine to clear inner heat and promote digestion. In Guangdong, some people will use LPA as a spice (11). These folk use records reflect: (1) LPA is relatively safe to eat and (2) LPA has adequate nutritional content.

**Figure 1:**
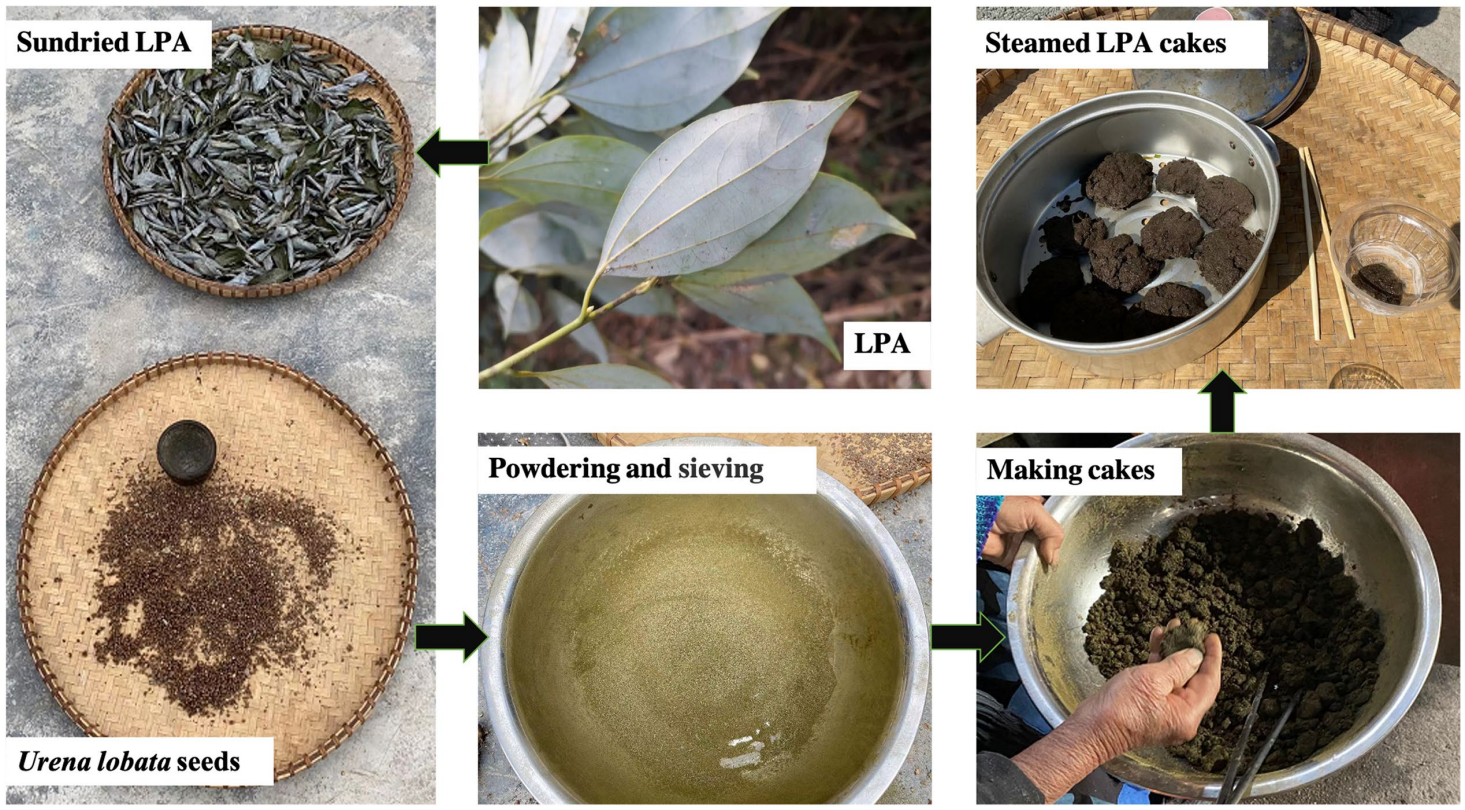
Process of making LPA cakes.

During the ethnobotanical survey in the Baiku Yao area of Nandan County, Guangxi, some local elders told us that they used LPA to make cakes (steam bread) during the famine years. Since LPA is an evergreen broad-leaved tree, it can be collected in different seasons. Therefore, we also participated in and recorded the production process of LPA cakes (see Figure 1). Locals collect LPA leaves from around the community, dry them in the sun, mash them into powders and sift them, then add a small amount of water to the sifted powder and stir it into a paste, then make a cake and steam it in a steamer to eat. If in autumn, after the *Urena lobata* seeds are mature, the local people will collect the seeds and dry them together, crush them to remove the shell, take out the seed kernels, grind them into powder, and add them to LPA powder, and then make them into cakes. *U. lobata* is a wild weed that can be seen everywhere in the local area, and its seeds are rich in oil (13). According to the local people, the powder of ground *U. lobata* seeds can increase the viscosity, better to improve the shape of the cake, and at the same time, improve the taste of LPA cakes.

In the Baiku Yao area, LPA is also the raw material for making local incense. Mainly dry and crush LPA leaves; then use thin bamboo sticks and LPA powder to press into incense. The jelly-like substance in LPA is said to allow the incense to hold its shape better, and its aromatic oils also make the incense more flammable and durable (“not easy to extinguish”). However, the “time-consuming and labor-intensive” local incense production process has gradually been forgotten with the modernization.

### 3.2. Nutrition components and total flavonoids of LPA

In order to verify the scientificity of traditional utilization knowledge of LPA, this study determined the contents of energy, protein, fat, carbohydrate, dietary fiber, minerals, amino acids, vitamins, and flavonoids in LPA leaves (results are shown in Table 2) (14, 15).

**Table 2:** The result of the nutritional analysis on LPA.

Test items		Test results	Reference intake amount daily (14, 15)
Macronutrient (g/100 g)	Energy	1,239 kJ (239 kcal)	–
	Protein	11.4	55 g/d ~ 65 g/d
	Fat	4	44 g/d ~ 78 g/d
	Soluble dietary fiber	0.903	–
	Dissoluble dietary fiber	43.5	–
	Total dietary fiber	44.4	27 g/d
	Starch	28.9	120 g/d ~ 150 g/d (carbohydrates)
Minerals (mg/100 g)	Cu	0.839	0.8 mg/d
	Fe	6.29	12–20 mg/d
	Zn	6.94	7.5–12.5 mg/d
	Ca	1,660	800 mg/d
	Mg	172	330 mg/d
	K	423	2000 mg/d
	Na	3.42	1,500 mg/d
	P	100	720 mg/d
Vitamins (mg/100 g)	Vitamin A	undetected	0.7–0.8 mg/d
	Vitamin B1	undetected	1.2–1.4 mg/d
	Vitamin B2	0.263	1.2–1.4 mg/d
	Vitamin C	17.8	100 mg/d
	Vitamin E	46.9	14 mg/d
Total flavonoids (mg/100 g)		770	–

#### 3.2.1. The macronutrients in LPA

LPA can provide 239 kcal/100 g of calories, which is much higher than other vegetables. For example, the edible portion of Dutch beans can only provide 27 kcal/100 g, and pumpkins can provide 22 kcal/100 g (16). In general, cereals have a high energetic value, with more than 300 kcal per 100 g. For example, wheat has 317 kcal/100 g and millet 358 kcal/100 g, while LPA is relatively low, indicating that its energy efficiency is slightly lower than other cereals (16). However, LPA has far more calories than most rhizome-type staple plants, such as potatoes (76 kcal/100 g), sweet potatoes (99 kcal/100 g), and cassava (116 kcal/100 g). The carbohydrate content of LPA is also outstanding, in which the starch content is 28.9 g/100 g, significantly higher than that of potato, but also lower than that of grains (16). Regarding energy and starch content, LPA is between potato and grain, which shows that it is scientific for local people to use it as food rations or pig feed in some areas during the famine.

LPA is rich in protein, 11.4 g per 100 g edible part, while the average protein content per 100 g eggs is about 13.3 g. Wheat is a good source of proteins, with protein content equal to 11.9 g/100 g, which is very similar to LPA, according to the data. Therefore, LPA as green leafy food, compared with eggs and wheat, has a rich protein content and can be used as one of the sources of human daily protein intake.

Dietary fiber, a class of non-starch polysaccharides in carbohydrates, has many health benefits, including reducing the risk of heart disease and type 2 diabetes, and is mainly found in vegetables, fruits, whole grains, and legumes (17). The total dietary fiber content of LPA is 44.4 g/100 g, most of which is insoluble dietary fiber, 43.5 g/100 g, and only 0.903 g/100 g is soluble dietary fiber. The total dietary fiber content of LPA is much higher than that of common cereals (16). For example, the total dietary fiber content of wheat is only 10.8 g/100 g, and that of bran is 31.3 g/100 g (16). The dietary fiber intake recommended in China is about 27 g per day (14), and less than 100 g of LPA can meet the daily dietary fiber requirements of the human body.

Generally speaking, LPA is very rich in energy substances, protein, and fiber, which can also meet the human body’s needs as a daily staple food. It is a plant with high potential economic value as a staple food worth further development.

#### 3.2.2. Trace elements content in LPA

Wild food plants have been an excellent source of micronutrients, and their consumption helps to meet nutritional requirements and overcome micronutrient deficiencies at a minimal cost (17, 18). In this study, the trace elements content of LPA was also determined, see Table 2. LPA is very rich in calcium. 50 g of LPA can meet the daily recommended calcium intake of the human body; LPA is also very rich in copper, and almost 100 g of LPA can meet the daily copper demand of the human body. In addition, the content of iron and zinc is also high, and 200 g of LPA can meet the daily iron and zinc requirements of the human body. Iron-rich spinach contains 2.9 mg of iron per 100 g of edible parts (16), while LPA has 6.94 mg/100 g; oysters can provide 9.39 mg/100 g of zinc, and beef have about 4 mg/100 g. The magnesium content of general nuts is relatively high, and almonds, in particular, have a magnesium content of about 178 mg per 100 g, while LPA has 172 mg of magnesium (16), which is nearly equivalent. The contents of potassium, sodium and phosphorus in LPA are relatively common. Generally speaking, LPA is rich in trace elements, especially calcium, copper, iron, zinc, and magnesium, which are difficult to obtain from general food, far more than other conventional vegetables, and can meet the needs of the human body with a small amount.

#### 3.2.3. Functional components in LPA

LPA is very low in vitamins A, B1, and B2. The vitamin C content is relatively good, at 17.8 mg/100 g, which is much higher than the vitamin C content of the super fruit blueberry (about 10 mg/100 g) (16). Vitamin E is a fat-soluble vitamin. Its main function is to act as an antioxidant to scavenge free radicals that may damage human cells, and it has the potential to promote health and prevent and treat diseases (20). LPA is very rich in vitamins, as high as 46.9 mg/100 g. Therefore, LPA is a very promising natural source of vitamin E.

Flavonoids exist in fruits and vegetables and have been proven to have many potential beneficial activities in animal studies and human trials, such as antioxidant, antibacterial, anti-cardiovascular disease, etc. (21). According to the test results, the flavonoid content of LPA is as high as 770 mg/100 g, which is much higher than that of ordinary vegetables and fruits (22, 23). This shows that LPA probably has very good dietary supplement potential and medicinal potential. However, this study only explored the content of total flavonoids and did not separate, extract and identify flavonoids. In future functional research on LPA, its flavonoid types still need to be fully identified.

## 4. Discussion

### 4.1. Security and future development of LPA

Through literature review and field investigation, it can be concluded that LPA has been used as a staple food in the past and can also be used as a feed. Our experiments show that LPA is rich in nutrients, and the traditional custom of using it as food and feed has certain scientific connotations. In addition, LPA’s high vitamin E and flavonoid content suggest that it has excellent dietary health and medicinal potential. According to the records of *Flora of China*, the bark of LPA is used for medicinal purposes. The principle and whether the leaves also have medicinal effects is worth further exploration.

Although the present study has provided a comprehensive analysis of the nutritional composition of LPA leave, the phytate content of LPA was not measured in this study. Phytates are known to bind with certain trace elements, such as iron and zinc, in the gut and reduce their bioavailability (24, 25). Therefore, measuring the phytate content of LPA is important to better understand the potential impact on trace element bioavailability. Future studies should aim to determine the phytate content of LPA, as well as its effect on the bioavailability of trace elements. Additionally, in order to estimate the potential dietary intake of LPA, *in vitro* digestion experiments should be conducted to simulate the digestive process and determine the bioaccessibility of nutrients in LPA (26). This would provide more accurate information on the nutritional value of LPA as a potential food source.

Additionally, the safety of the kernel of *U. lobata*, which is the auxiliary material of LPA cake, has not been verified. For example, Ahmad et al. isolated and identified malvalic acid and sterculic acid in *U. lobata* seed oil, and both were proved to be somewhat toxic (13). However, research on the composition of LPA is still rare; only Huang et al. have studied its volatile components (27). This study shows that LPA has high volatile oil content and pleasing aroma so that it can be developed and planted as a natural spice, and it also reflects the scientific nature of the tradition of using it as a spice in some areas. However, there is no research on the safety and dose of toxic components of LPA. If it is to be developed into a new type of food or feed in the future, toxicity research is essential. Due to its rich aroma and high fiber content, LPA does not taste good as a food, and it was used to satisfy hunger during famines in the past. Therefore, whether as food or feed, improving its smell and palatability is also necessary. With further research, LPA could be developed into a thriving industry, driving economic growth in local communities.

### 4.2. Traditional knowledge under social change

Using LPA to make cake has very strong characteristics of the times, which intuitively shows that the relevant traditional knowledge is gradually disappearing. The information reporters who know this traditional usage are all older adults over the age of seventy, and the next generation of local people expressed complete ignorance of this knowledge. With the accelerated pace of social change and modernization, more and more traditional knowledge with the characteristics of the times will disappear. The related biological and cultural diversity will gradually decrease, so recording and saving them becomes increasingly important. Baiku Yao, as a branch of ethnic minorities that “passed directly from primitive society to modern society”, has relatively perfect preservation of its traditional knowledge, and it is very worthy of our urgent rescue. Therefore, the next step of our team is to carry out systematic research on the traditional edible plants of Baiku Yao.

Under the background of “modernization threatens traditional knowledge”, traditional knowledge has been paid more and more attention in recent years. In particular, the global COVID-19 pandemic at the end of 2019, frequent extreme weather, and a series of recent international geopolitical wars led to the disintegration of highly integrated world trade, resulting in food and material crises worldwide (28). Therefore, in this kind of survival crisis, the use of wild medicinal plants and edible plants began to be picked up again. Using and managing wild plant resources can effectively enhance people’s ability to resist natural and man-made disasters (29). This new global crisis gives ethnobotany and ethnic biology a new era’s significance and mission (29). In addition, biodiversity protection is a consensus reached by everyone in the past. In a social-ecological system, biodiversity and cultural diversity are intertwined and inseparable. Thus, to protect traditional knowledge is to protect cultural diversity and biodiversity (30).

## 5. Conclusion

This research studies the folk utilization of LPA plants. According to the survey, LPA can be eaten as a staple food in the Baiku Yao area, and it is also used as a spice and feed in other areas. The test results of the nutritional components of LPA show that: the plant contains high starch, protein, and dietary fiber; the plant is also rich in trace elements, vitamin E, and total flavonoids. The traditional knowledge of LPA as a staple food and feedstuff has some scientific basis. LPA has the economic potential to be developed into a new type of food or feed.

## Data availability statement

The original contributions presented in the study are included in the article/supplementary material, further inquiries can be directed to the corresponding author.

## Author contributions

BL and RH contributed to the conception and design of the study. RH and YN performed the data collection and statistical analysis. BL wrote the first draft of the manuscript. All authors contributed to the article and approved the submitted version.

## Funding

This study has been supported by the National Natural Science Foundation of China (32000264); Survey and Collection of Germplasm Resources of Woody & Herbaceous Plants in Guangxi, China (GXFS-2021-34), and Guangxi Chinese medicine key disciplines construction projects (GZXK-Z-20-69).

## Conflict of interest

The authors declare that the research was conducted in the absence of any commercial or financial relationships that could be construed as a potential conflict of interest.

## Publisher’s note

All claims expressed in this article are solely those of the authors and do not necessarily represent those of their affiliated organizations, or those of the publisher, the editors and the reviewers. Any product that may be evaluated in this article, or claim that may be made by its manufacturer, is not guaranteed or endorsed by the publisher.
